# Coordinated DNA Replication by the Bacteriophage T4 Replisome

**DOI:** 10.3390/v7062766

**Published:** 2015-06-19

**Authors:** Erin Noble, Michelle M. Spiering, Stephen J. Benkovic

**Affiliations:** Pennsylvania State University, Department of Chemistry, 414 Wartik Laboratory, University Park, PA 16802, USA; E-Mails: eun2@psu.edu (E.N.); mms36@psu.edu (M.M.S.)

**Keywords:** DNA replication, T4 bacteriophage, replisome

## Abstract

The T4 bacteriophage encodes eight proteins, which are sufficient to carry out coordinated leading and lagging strand DNA synthesis. These purified proteins have been used to reconstitute DNA synthesis *in vitro* and are a well-characterized model system. Recent work on the T4 replisome has yielded more detailed insight into the dynamics and coordination of proteins at the replication fork. Since the leading and lagging strands are synthesized in opposite directions, coordination of DNA synthesis as well as priming and unwinding is accomplished by several protein complexes. These protein complexes serve to link catalytic activities and physically tether proteins to the replication fork. Essential to both leading and lagging strand synthesis is the formation of a holoenzyme complex composed of the polymerase and a processivity clamp. The two holoenzymes form a dimer allowing the lagging strand polymerase to be retained within the replisome after completion of each Okazaki fragment. The helicase and primase also form a complex known as the primosome, which unwinds the duplex DNA while also synthesizing primers on the lagging strand. Future studies will likely focus on defining the orientations and architecture of protein complexes at the replication fork.

## 1. Introduction

**Figure 1:**
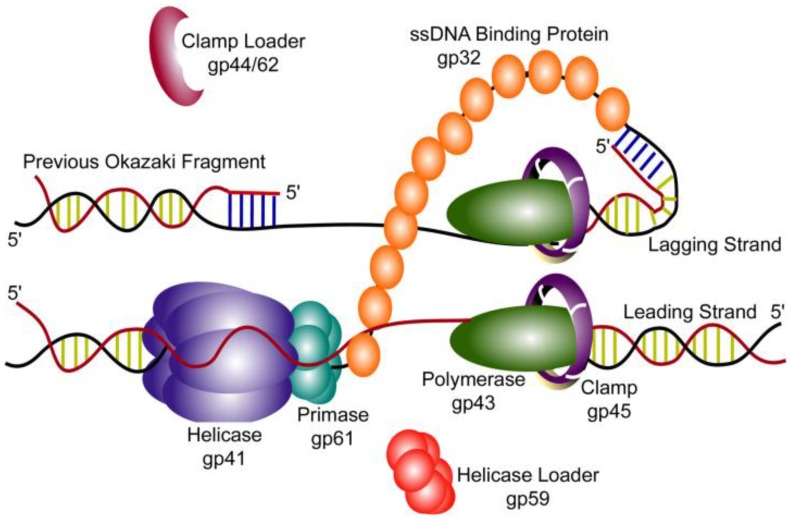
A model of the T4 bacteriophage DNA replisome. Replication of T4 genomic DNA is accomplished by a replication complex composed of eight proteins. The helicase (gp41) and primase (gp61) interact to form the primosome with the assistance of the helicase loader (gp59). The primosome complex encircles the lagging strand DNA, unwinding duplex DNA while synthesizing RNA primers for use by the lagging strand polymerase (gp43). DNA synthesis on both strands is catalyzed by a holoenzyme complex formed by a polymerase (gp43) and a trimeric processivity clamp (gp45). The clamp is loaded onto the DNA by the clamp loader complex (gp44/62). The leading and lagging strand holoenzymes interact to form a dimer. Single-stranded DNA formed by the helicase is coated with single-stranded DNA-binding protein (gp32).

Bacteriophages were first discovered in the early 20th century due to their ability to kill bacteria [1]. Apart from their therapeutic uses, bacteriophages were found to encode proteins that carried out many of the same basic processes that are found in eukaryotic cells. The T4 bacteriophage, which infects *Escherichia coli*, is one of the best-studied viruses in this group. Its double-stranded DNA genome encodes all of the proteins necessary to carry out viral DNA replication in the infected cell. The components of the T4 replisome can be purified and used to reconstitute DNA replication *in vitro*. This system has been well characterized as a model for DNA replication at a fork [2,3,4]. The T4 replisome consists of eight proteins, which together catalyze coordinated leading and lagging strand synthesis (Figure 1). These proteins are similar in structure and function to their eukaryotic homologues [5]. Studies on the T4 system have contributed greatly to the understanding of DNA replication and paved the way for current studies on human and yeast DNA replication. This review will cover the current understanding of T4 DNA replication and highlight areas where recent research has yielded new mechanistic insight into functioning of the T4 replisome. For more detail on other prokaryotic model systems, see recent reviews highlighting studies of the T7 bacteriophage and *E. coli* replisomes [6,7,8].

## 2. T4 Replication Fork Components

T4 replication can be initiated via several different pathways [9]. Two specialized structures, R-loops and D-loops, have been shown to be important. R-loops form at T4 origin sites where an RNA primer is synthesized. D-loops are formed by the recombination machinery and are used to initiate origin-independent DNA synthesis. These two mechanisms of DNA replication initiation of have been reviewed elsewhere [10].

Synthesis of the T4 genomic DNA is accomplished by a holoenzyme complex composed of the gp43 polymerase and the gp45 sliding clamp [11,12,13]. On the leading strand, DNA synthesis is carried out continuously by one holoenzyme complex. On the lagging strand, DNA is synthesized in the opposite direction of the progression of the replication fork. Multiple priming events allow a second holoenzyme complex to carry out DNA synthesis discontinuously in 1 to 2 kb fragments known as Okazaki fragments. While there is no available crystal structure for the T4 gp43, the structure for the RB69 bacteriophage gp43 has been solved alone and as part of a binary and ternary complex [14,15,16]. The two proteins are 62% identical and 74% similar and thus, the proteins are likely very similar in topology. The RB69 structure reveals five conserved domains in a configuration similar to that of the eukaryotic B family polymerases. The *N*-terminus contains a 3′ to 5′ exonuclease active site. This truncated exonuclease domain from T4 gp43 has been isolated and the structure solved [12]. The catalytic activity of this domain is independent from the rest of the polymerase, as it retains full exonuclease activity *in vitro* [17]. The *C*-terminus of RB69 gp43 is organized into conserved finger, palm, and thumb domains, which catalyze DNA polymerization 5′ to 3′ [15].

The T4 sliding clamp, gp45, is a ring-shaped, trimeric protein that serves as a processivity factor for the polymerase [18,19]. The inner diameter of the ring is about 35 Å, which is large enough to accommodate duplex DNA. Unlike clamps in other systems, the T4 clamp exists in solution as a partially open ring with one of the three subunit interfaces disrupted [20,21,22]. Once loaded onto DNA, the interior of the clamp interacts with the DNA phosphate backbone through a number of basic residues and anchors the polymerase to the DNA [19]. gp43 has a C-terminal PIP box domain that mediates the interaction of the polymerase and the sliding clamp [23].

The circular gp45 clamp is loaded onto the DNA by a clamp loader complex. In T4, four gp44 subunits associate with one gp62 subunit forming the gp44/62 clamp loader [24]. Each gp44 subunit binds ATP and the complex has a strong DNA-dependent ATPase activity [25,26]. The clamp loader is a member of the AAA+ family of ATPases, but unlike other enzymes of this type, clamp loaders are pentameric rather than hexameric. This asymmetry results in a gap that allows the clamp loader to specifically recognize the primer-template junction when loading a clamp [27,28].

The T4 helicase, gp41, forms a hexamer upon binding GTP or ATP [29]. This active form of the helicase hydrolyzes GTP/ATP to move along single-stranded DNA [30,31]. Electron microscopy has revealed that there are two forms of the hexameric gp41, a symmetric ring and a gapped asymmetric ring [32]. The “open” ring is thought to be important for the loading of the helicase onto DNA [29]. As part of the replication fork, gp41 unwinds the double stranded DNA by traveling 5′ to 3′, encircling the lagging strand while excluding the leading strand [33]. The preferred substrate for the helicase is a forked DNA with both 5′ and 3′ single-stranded DNA regions, suggesting the protein interacts with both the leading and lagging strands [33,34]. T4 also encodes two other helicases, UvsW and Dda. Both accessory helicases have been suggested to have roles in replication initiation, recombination, and repair (see review [35]).

Priming on the lagging strand is catalyzed by the gp61 primase, which interacts with gp41 to form the primosome [36]. This primosome synthesizes pentaribonucleotides from 5′-GTT-3′ priming sites. The 3′-T is necessary for priming but is not used to template the primer; the resulting primers have the sequence 5′-pppACNNN-3′ [37]. At high concentrations *in vitro*, gp61 alone can synthesize some RNA primers, but they are typically dimers primed from a 5′-GCT-3′ site [37,38]. In the presence of gp41, the rate of primer synthesis increases and shifts to pentaribonucleotide products primed from 5′-GTT-3′ sites, which is the priming site used *in vivo* [38,39]. gp61 alone is monomeric, but in the presence of gp41 and/or DNA, it oligomerizes into a hexameric ring [32,40].

Exposed single-stranded DNA is bound by gp32, which is necessary for DNA replication *in vivo*. It has many functions including preventing the formation of DNA secondary structure, protecting DNA from nuclease digestion, and stimulation of the gp43 synthesis rate and processivity [41,42,43]. A crystal structure of gp32 in complex with DNA reveals three domains. The N-terminus binds other gp32 monomers allowing for oligomerization, the *C*-terminus mediates interactions with other proteins such as the T4 polymerase, and the core domain binds single-stranded DNA [44].

*In vivo* a helicase loader, gp59, is required for origin-dependent initiation of replication [45]. In the presence of gp32, the helicase cannot efficiently load onto the DNA fork without the addition of gp59. gp59 interacts with gp41 stoichiometrically and helps to displace gp32, allowing the helicase to load [46]. gp59 is thought to mediate loading by inducing a conformational change in gp41 that promotes DNA binding [47]. It is unclear if gp59 dissociates or remains as part of the replication complex [48,49]. Binding events between gp43 and gp59 have been observed using single-molecule FRET [50].

## 3. Holoenzyme Formation

The gp43 polymerase alone can only copy short stretches of single-stranded DNA without dissociating [51]. The gp45 sliding clamp is a homotrimeric ring that allows gp43 to catalyze processive DNA synthesis. It is loaded onto DNA by gp44/62 with the clamp loader specifically recognizing the free 3′ end of the primer-template junction. As the clamp is partially open in solution, the function of the T4 clamp loader is to stabilize the open clamp and direct it onto DNA in the correct orientation. Crystal structures of the clamp/clamp loader complex, both with and without DNA, have provided detailed insight into how loading occurs [52]. The clamp loader has a low affinity for the clamp until the binding of ATP through an AAA+ module in each of the gp44 subunits. ATP binding causes the clamp loader subunits to adopt a spiral conformation that can bind to the clamp and open it further, allowing it to be loaded onto DNA. The opening of the clamp occurs in two planes. Movement of ~9 Å in the plane of the ring allows single-stranded DNA to pass through the gap, while an out-of-plane shift of ~23 Å results in a twisted conformation of the clamp, aligning it with the helical structure of the DNA. DNA binding stimulates the ATPase activity of the clamp loader and the hydrolysis of ATP in each of the four gp44 subunits [24,53]. This hydrolysis triggers a change in the conformation of the clamp loader, which closes the clamp around the DNA.

Once the clamp is closed around the DNA, it must be bound by the polymerase to form the holoenzyme. This process has been characterized using a FRET-based assay to monitor clamp loading and holoenzyme assembly. The clamp and clamp loader complex rapidly bind to the DNA after ATP binding. In the absence of the gp43 polymerase, the clamp and clamp loader remain as a complex and dissociate from the DNA together. In the presence of the polymerase, a functional holoenzyme forms in three kinetically distinct steps. The first corresponds to the hydrolysis of ATP and the dissociation of the clamp loader. The subsequent two steps involve slower conformational changes leading to the formation of a stable complex. The dissociation of the clamp in the presence of the polymerase is significantly slower than the clamp alone [54]. This stable holoenzyme complex is then able to efficiently carry out processive DNA synthesis on the leading strand and discontinuous DNA synthesis on the lagging strand.

## 4. Holoenzyme Processivity

The holoenzyme on the leading strand synthesizes DNA in the same direction as the movement of the replication fork. *In vivo*, the T4 genome can be synthesized within 15 min [55]. The half-life of the holoenzyme complex has been measured as 11 min as part of a moving fork and about 6 min on a small, defined DNA fork structure [56,57]. Given the half-life of the holoenzyme and the speed of synthesis, it is possible that the entire T4 genome could be synthesized by a single holoenzyme on the leading strand. While this highly processive holoenzyme would be advantageous on the leading strand, the lagging strand is synthesized discontinuously and the holoenzyme must repeatedly dissociate and rebind for synthesis of each Okazaki fragment.

A more recent study probing the processivity of the T4 holoenzyme confirmed the long half-life during replication using a standard dilution experiment [58]. However, it was found that an inactive mutant of the polymerase (D408N) was able to rapidly displace the wild-type polymerase and inhibit DNA synthesis. This inhibition occurred on both the leading and lagging strands. These results suggest that although the polymerase will not readily dissociate on its own, it can be actively displaced by a second polymerase without affecting DNA synthesis. The exchange process was termed dynamic processivity and is thought to be mediated through interactions with gp45 [58]. The *C*-terminus of gp43 is essential for polymerase binding to the clamp, but its deletion does not affect DNA polymerization [23]. When polymerase containing this deletion was used as a trap, it could no longer displace the replicating polymerase [58]. As the clamp is trimeric, it is hypothesized that multiple polymerases could bind and facilitate the exchange. This “toolbelt” model for the clamp has been suggested in other systems as well, with numerous proteins involved in DNA replication and repair also containing clamp binding domains [59,60]. In the T7 system, where there is no sliding clamp, the exchange process has been shown to be mediated by an interaction between the polymerase and the helicase [61]. It is thought that the helicase can bind multiple polymerases facilitating exchange on the leading strand and recycling on the lagging strand.

## 5. Coupling of Helicase and Polymerase for Leading Strand Synthesis

While both gp41 helicase and gp43/gp45 holoenzyme can function independently *in vitro* to unwind duplex DNA, the two enzymes work best when their activities are combined. The helicase alone is significantly slower and less processive than the replication fork, and the holoenzyme is very inefficient at strand displacement synthesis [33,62]. Together, the helicase and holoenzyme are able to efficiently carry out leading strand synthesis [63]. In the presence of a macromolecular crowding reagent, only gp43 and gp41 are needed, indicating the clamp does not play a role [64]. While the functional coupling between the two proteins has been clearly demonstrated, there is no evidence of a physical interaction between gp43 and gp41 [65,66]. One study also found that the T4 polymerase could be replaced with another processive polymerase and still carry out strand displacement synthesis, but could not be replaced with a low processivity polymerase [65]. This suggests that each enzyme is stabilized on the DNA replication fork by the activity of the other, with the helicase providing single-stranded DNA that the polymerase then traps.

In the T7 system, it was reported that nucleotide incorporation by the polymerase provided the driving force to stimulate helicase activity, but a detailed mechanism for helicase-polymerase coupling was not described [67]. A more recent single-molecule study of the coupling in the T4 system used magnetic tweezers to monitor both coupled and uncoupled activity [68]. A DNA hairpin was tethered to a glass slide with a magnetic bead on the other end. Force was applied to destabilize the duplex and assist enzymes in opening the hairpin. At low force, where the duplex of the hairpin is stable, the helicase moved at 6 times slower than its maximal translocation rate and showed sequence dependent pausing. As higher force was applied, the rate of helicase activity increased dramatically. Additionally, at low helicase concentrations, significant helicase slippage was observed involving the reannealing of tens to hundreds of base pairs. This fits with the passive model of helicase activity previously demonstrated, in which the helicase is not efficient in destabilizing duplex DNA and relies on transient fraying of base pairs to move forward [69].

The T4 holoenzyme was found to have very low strand displacement activity at low force and mainly exhibited exonuclease activity [68]. When higher forces destabilized the duplex, the holoenzyme was able to replicate the hairpin at maximal speeds. At moderate forces, the holoenzyme exhibited pausing and stalling. The proportion of holoenzymes observed synthesizing DNA, pausing, or degrading DNA was highly dependent on the force used. This indicates that at higher forces the holoenzyme is able to stay in the polymerization mode, while lower forces shift the holoenzyme to the exonuclease mode. When pausing and exonuclease events were excluded from analysis, the holoenzyme activity fits with a model of a strongly active motor. The basis for collaborative coupling then emerges in a model where the helicase provides the single-stranded DNA for the holoenzyme, but also prevents the fork regression pressure from switching the polymerase into the exonuclease mode. As the holoenzyme is kept in its highly processive polymerization mode, it stimulates the activity of the helicase and prevents slippage backwards [68].

## 6. Coordination of Helicase and Priming on the Lagging Strand

The leading and lagging strands are thought to be synthesized at the same net rate, despite the need for repeated priming and extension events on the lagging strand [4,70,71]. Priming is catalyzed by a gp61-gp41 complex known as the primosome. Both priming and DNA unwinding activity are stimulated when both proteins are present [34,38,39]. There is strong biochemical evidence for the interaction of the hexameric gp41 helicase and oligomeric gp61 primase [34,36,72,73]. Importantly, a gp61-gp41 fusion protein has been shown to have close to wild-type priming and helicase activity and can successfully catalyze coordinated leading and lagging strand synthesis [74].

This tight coordination of activity is clear, despite the fact that the helicase travels 5′ to 3′ unwinding duplex DNA while the primase synthesizes primers 3′ to 5′ on the same strand. There are three models for how this coupling can occur. The first model suggests that the helicase, and possibly the whole replisome, pauses while the primers are being synthesized. In the second model, primase subunits dissociate from the helicase and are left behind to synthesize primers. In the third model, coupling is accomplished by the formation of priming loops wherein the lagging strand folds back allowing for priming. The loop is then released after the primer is synthesized.

**Figure 2:**
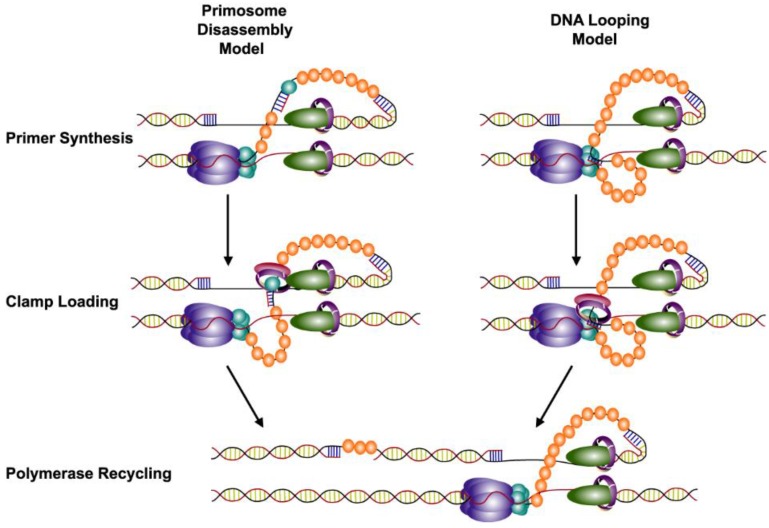
The two models of primosome activity used by T4 to initiate lagging strand synthesis. The helicase (gp41) and primase (gp61) interact as stacked rings encircling the lagging strand. This complex unwinds duplex DNA while synthesizing pentaribonucleotide RNA primers for use by the lagging strand polymerase (gp43). Primer synthesis occurs while the helicase continues to unwind DNA in the opposite direction. Two models have been proposed to accommodate these coupled activities. In the primosome disassembly model (shown left), one of the primase subunits dissociates from the primosome complex and remains with the newly synthesized primer. In the DNA looping model (shown right), the excess DNA unwound by the helicase during primer synthesis loops out allowing the primase to stay intact. In both models, the clamp loader (gp44/62) loads a clamp (gp45) onto the newly synthesized primer. The lagging strand polymerase is then signaled to release and recycle to the new primer.

By observing helicase and priming activity on DNA hairpins using magnetic tweezers, the role of the three models in the T4 primosome could be directly observed [74]. In the T7 system, both pausing of the primosome [75] and priming loops have been reported [76]. The T4 study yielded no evidence of pausing of the T4 primosome. However, clear evidence of both primase disassembly and looping were seen in these experiments, indicating that there are two different mechanisms used by T4 to couple the helicase and primase (Figure 2). While primase disassembly was the predominant mode, in the case where the primase and helicase were fused only the looping mechanism was seen.

## 7. Recycling of the Lagging Strand Polymerase

The trombone model was proposed to explain the coordination of leading and lagging strand synthesis with the two polymerases synthesizing in opposite directions. In this model, the lagging strand DNA loops out during the formation of each Okazaki fragment [4]. These loops have been visualized in electron micrographs of T4 replication products [48]. The lagging strand polymerase is retained as part of the replisome after completing synthesis of each Okazaki fragment [4]. It dissociates from the DNA, but then rapidly binds the next primer to continue synthesis. This recycling of the lagging strand polymerase is supported by numerous studies. While the clamp, clamp loader, primase, and gp32 have all been shown to exchange with proteins in solution during replication, the polymerase is resistant to dilution [77,78,79,80]. The size of the Okazaki fragments is also independent of polymerase concentration [4,58]. Importantly, the leading and lagging strand polymerases interact in the presence of DNA, which provides a mechanism for tethering the lagging strand polymerase to the replisome [66].

While the holoenzyme on the leading strand is highly processive, on the lagging strand it must repeatedly dissociate. The trigger for the dissociation of the lagging strand polymerase has not clearly been defined despite a number of studies. Several models have been proposed with two gaining the most support and evidence suggests that both play a role during replication [81]. The collision model proposes that the lagging strand polymerase dissociates after colliding with the end of the previous Okazaki fragment, and this stimulates the primase to synthesize a new primer [62,82]. However, it has been also shown that dissociation of the lagging strand polymerase can occur before reaching the previous Okazaki fragment leaving single-stranded DNA gaps [81]. To account for this observation, the signaling model has been proposed where recycling is triggered by the synthesis of a new primer and the timing controlled by gp61 [80,81,83]. Recently, additional signals have been proposed to regulate this recycling in other replication systems such *E. coli* and T7. These new triggers include tension induced dissociation of the polymerase [84], primer availability [85], and a third polymerase [86]. While it has been shown that a third T4 polymerase does not seem to play a role in Okazaki fragment synthesis [87], the nature of the signal for recycling is still unknown.

## 8. Future Directions

The major unanswered questions concerning T4 DNA replication involve understanding the dynamics and organization of the proteins at the replication fork. While the protein complexes involved in replication, the primosome, holoenzyme, and single-stranded DNA binding protein, have been extensively studied, their orientations and spatial juxtapositions at the fork are unclear. According to the trombone model, the two polymerases are thought to interact in opposite orientations, but how these proteins assemble at the fork has not been demonstrated. It is also not known how the polymerases at the replication fork are able to readily exchange with polymerases in solution. It is possible that the polymerase and clamp transiently separate yielding the dynamic processivity that has been observed. Another area of uncertainty is the trigger for recycling of the lagging strand polymerase. A number of possible signals have been suggested but none of these models have been proven. Fluorescence resonance energy transfer (FRET) and single-molecule experiments will likely play an important role in resolving these uncertainties and more clearly defining the organization and coordination of the T4 replisome.

## References

[1] Sulakvelidze A., Alavidze Z., Morris J.G. (2001). Bacteriophage therapy. Antimicrob. Agents Chemother..

[2] Nossal N.G. (1992). Protein-protein interactions at a DNA replication fork: Bacteriophage T4 as a model. FASEB J..

[3] Liu C., Burke R., Hibner U., Barry J., Alberts B. (1979). Probing DNA Replication Mechanisms with the T4 Bacteriophage *in Vitro* System. Cold Spring Harbor Symposia on Quantitative Biology.

[4] Alberts B., Barry J., Bedinger P., Formosa T., Jongeneel C., Kreuzer K. (1983). Studies on DNA Replication in the Bacteriophage T4 *in Vitro* System. Cold Spring Harbor Symposia on Quantitative Biology.

[5] Mueser T.C., Hinerman J.M., Devos J.M., Boyer R.A., Williams K.J. (2010). Structural analysis of bacteriophage T4 DNA replication: A review in the virology journal series on bacteriophage T4 and its relatives. Virol. J..

[6] Lee S.-J., Richardson C.C. (2011). Choreography of bacteriophage T7 DNA replication. Curr. Opin. Chem. Biol..

[7] Hamdan S.M., Richardson C.C. (2009). Motors, switches, and contacts in the replisome. Annu. Rev. Biochem..

[8] Van Oijen A.M., Loparo J.J. (2010). Single-molecule studies of the replisome. Annu. Rev. Biophys..

[9] Mosig G., Colowick N., Gruidl M.E., Chang A., Harvey A.J. (1995). Multiple initiation mechanisms adapt phage T4 DNA replication to physiological changes during T4’s development. FEMS Microb. Rev..

[10] Kreuzer K.N., Brister J.R. (2010). Initiation of bacteriophage T4 DNA replication and replication fork dynamics: A review in the virology journal series on bacteriophage T4 and its relatives. Virol. J..

[11] Benkovic S.J., Valentine A.M., Salinas F. (2001). Replisome-mediated DNA replication. Annu. Rev. Biochem..

[12] Reddy M.K., Weitzel S.E., von Hippel P.H. (1993). Assembly of a functional replication complex without ATP hydrolysis: A direct interaction of bacteriophage T4 gp45 with T4 DNA polymerase. Proc. Natl. Acad. Sci. USA.

[13] Sexton D.J., Berdis A.J., Benkovic S.J. (1997). Assembly and disassembly of DNA polymerase holoenzyme. Curr. Opin. Chem. Biol..

[14] Franklin M.C., Wang J., Steitz T.A. (2001). Structure of the replicating complex of a pol α family DNA polymerase. Cell.

[15] Wang J., Sattar A.A., Wang C., Karam J., Konigsberg W., Steitz T. (1997). Crystal structure of a pol α family replication DNA polymerase from bacteriophage RB69. Cell.

[16] Shamoo Y., Steitz T.A. (1999). Building a replisome from interacting pieces: Sliding clamp complexed to a peptide from DNA polymerase and a polymerase editing complex. Cell.

[17] Lin T.-C., Karam G., Konigsberg W.H. (1994). Isolation, characterization, and kinetic properties of truncated forms of T4 DNA polymerase that exhibit 3′–5′exonuclease activity. J. Biol. Chem..

[18] Bruck I., O’Donnell M. (2001). The ring-type polymerase sliding clamp family. Genome Biol..

[19] Moarefi I., Jeruzalmi D., Turner J., O’Donnell M., Kuriyan J. (2000). Crystal structure of the DNA polymerase processivity factor of T4 bacteriophage. J. Mol. Biol..

[20] Soumillion P., Sexton D.J., Benkovic S.J. (1998). Clamp subunit dissociation dictates bacteriophage T4 DNA polymerase holoenzyme disassembly. Biochemistry.

[21] Alley S.C., Shier V.K., Abel-Santos E., Sexton D.J., Soumillion P., Benkovic S.J. (1999). Sliding clamp of the bacteriophage T4 polymerase has open and closed subunit interfaces in solution. Biochemistry.

[22] Millar D., Trakselis M.A., Benkovic S.J. (2004). On the solution structure of the T4 sliding clamp (gp45). Biochemistry.

[23] Berdis A.J., Soumillion P., Benkovic S.J. (1996). The carboxyl terminus of the bacteriophage T4 DNA polymerase is required for holoenzyme complex formation. Proc. Natl. Acad. Sci. USA.

[24] Jarvis T., Paul L., Hockensmith J., von Hippel P. (1989). Structural and enzymatic studies of the T4 DNA replication system. II. Atpase properties of the polymerase accessory protein complex. J. Biol. Chem..

[25] Jarvis T., Newport J., von Hippel P. (1991). Stimulation of the processivity of the DNA polymerase of bacteriophage T4 by the polymerase accessory proteins. The role of atp hydrolysis. J. Biol. Chem..

[26] Rush J., Lin T., Quinones M., Spicer E., Douglas I., Williams K., Konigsberg W. (1989). The 44p subunit of the T4 DNA polymerase accessory protein complex catalyzes ATP hydrolysis. J. Biol. Chem..

[27] Bowman G.D., O’Donnell M., Kuriyan J. (2004). Structural analysis of a eukaryotic sliding DNA clamp-clamp loader complex. Nature.

[28] Simonetta K.R., Kazmirski S.L., Goedken E.R., Cantor A.J., Kelch B.A., McNally R., Seyedin S.N., Makino D.L., O’Donnell M., Kuriyan J. (2009). The mechanism of ATP-dependent primer-template recognition by a clamp loader complex. Cell.

[29] Dong F., Gogol E.P., von Hippel P.H. (1995). The phage T4-coded DNA replication helicase (gp41) forms a hexamer upon activation by nucleoside triphosphate. J. Biol. Chem..

[30] Young M.C., Schultz D.E., Ring D., von Hippel P.H. (1994). Kinetic parameters of the translocation of bacteriophage T4 gene 41 protein helicase on single-stranded DNA. J. Mol. Biol..

[31] Liu C., Alberts B. (1981). Characterization of the DNA-dependent gtpase activity of T4 gene 41 protein, an essential component of the t4 bacteriophage DNA replication apparatus. J. Biol. Chem..

[32] Norcum M.T., Warrington J.A., Spiering M.M., Ishmael F.T., Trakselis M.A., Benkovic S.J. (2005). Architecture of the bacteriophage T4 primosome: Electron microscopy studies of helicase (gp41) and primase (gp61). Proc. Natl. Acad. Sci. USA.

[33] Venkatesan M., Silver L., Nossal N. (1982). Bacteriophage T4 gene 41 protein, required for the synthesis of RNA primers, is also a DNA helicase. J. Biol. Chem..

[34] Richardson R.W., Nossal N. (1989). Characterization of the bacteriophage T4 gene 41 DNA helicase. J. Biol. Chem..

[35] Perumal S.K., Raney K.D., Benkovic S.J. (2010). Analysis of the DNA translocation and unwinding activities of T4 phage helicases. Methods.

[36] Zhang Z., Spiering M.M., Trakselis M.A., Ishmael F.T., Xi J., Benkovic S.J., Hammes G.G. (2005). Assembly of the bacteriophage T4 primosome: Single-molecule and ensemble studies. Proc. Natl. Acad. Sci. USA.

[37] Cha T., Alberts B. (1986). Studies of the DNA helicase-RNA primase unit from bacteriophage T4. A trinucleotide sequence on the DNA template starts rna primer synthesis. J. Biol. Chem..

[38] Hinton D., Nossal N. (1987). Bacteriophage T4 DNA primase-helicase. Characterization of oligomer synthesis by T4 61 protein alone and in conjunction with T4 41 protein. J. Biol. Chem..

[39] Cha T.A., Alberts B.M. (1990). Effects of the bacteriophage T4 gene 41 and gene 32 proteins on rna primer synthesis: The coupling of leading-and lagging-strand DNA synthesis at a replication fork. Biochemistry.

[40] Yang J., Xi J., Zhuang Z., Benkovic S.J. (2005). The oligomeric T4 primase is the functional form during replication. J. Biol. Chem..

[41] Huberman J.A., Kornberg A., Alberts B.M. (1971). Stimulation of t4 bacteriophage DNA polymerase by the protein product of T4 gene 32. J. Mol. Biol..

[42] Huang C., Hearst J., Alberts B. (1981). Two types of replication proteins increase the rate at which T4 DNA polymerase traverses the helical regions in a single-stranded DNA template. J. Biol. Chem..

[43] Huang C.-C., Hearst J.E. (1980). Pauses at positions of secondary structure during *in vitro* replication of single-stranded fd Bacteriophage DNA by T4 DNA polymerase. Anal. Biochem..

[44] Shamoo Y., Friedman A.M., Parsons M.R., Konigsberg W.H., Steitz T.A. (1995). Crystal structure of a replication fork single-stranded DNA binding protein (T4 gp32) complexed to DNA. Nature.

[45] Dudas K.C., Kreuzer K.N. (2005). Bacteriophage T4 helicase loader protein gp59 functions as gatekeeper in origin-dependent replication *in vivo*. J. Biol. Chem..

[46] Ishmael F.T., Alley S.C., Benkovic S.J. (2002). Assembly of the Bacteriophage T4 helicase architecture and stoichiometry of the gp41-gp59 complex. J. Biol. Chem..

[47] Delagoutte E., von Hippel P.H. (2005). Mechanistic studies of the t4 DNA (gp41) replication helicase: Functional interactions of the c-terminal tails of the helicase subunits with the T4 (gp59) helicase loader protein. J. Mol. Biol..

[48] Chastain P.D., Makhov A.M., Nossal N.G., Griffith J. (2003). Architecture of the replication complex and DNA loops at the fork generated by the bacteriophage t4 proteins. J. Biol. Chem..

[49] Nossal N.G., Makhov A.M., Chastain P.D., Jones C.E., Griffith J.D. (2007). Architecture of the Bacteriophage T4 replication complex revealed with nanoscale biopointers. J. Biol. Chem..

[50] Zhao Y., Chen D., Yue H., Spiering M.M., Zhao C., Benkovic S.J., Huang T.J. (2014). Dark-field illumination on zero-mode waveguide/microfluidic hybrid chip reveals T4 replisomal protein interactions. Nano Lett..

[51] Mace D.C., Alberts B.M. (1984). T4 DNA polymerase: Rates and processivity on single-stranded DNA templates. J. Mol. Biol..

[52] Kelch B.A., Makino D.L., O’Donnell M., Kuriyan J. (2011). How a DNA polymerase clamp loader opens a sliding clamp. Science.

[53] Berdis A.J., Benkovic S.J. (1996). Role of adenosine 5′-triphosphate hydrolysis in the assembly of the bacteriophage T4 DNA replication holoenzyme complex. Biochemistry.

[54] Perumal S.K., Ren W., Lee T.-H., Benkovic S.J. (2013). How a holoenzyme for DNA replication is formed. Proc. Natl. Acad. Sci. USA.

[55] Mathews C.K. (1983). Bacteriophage T4.

[56] Kaboord B.F., Benkovic S.J. (1995). Accessory proteins function as matchmakers in the assembly of the T4 DNA polymerase holoenzyme. Curr. Biol..

[57] Schrock R.D., Alberts B. (1996). Processivity of the gene 41 DNA helicase at the bacteriophage T4 DNA replication fork. J. Biol. Chem..

[58] Yang J., Zhuang Z., Roccasecca R.M., Trakselis M.A., Benkovic S.J. (2004). The dynamic processivity of the T4 DNA polymerase during replication. Proc. Natl. Acad. Sci. USA.

[59] Maga G., Hübscher U. (2003). Proliferating cell nuclear antigen (PCNA): A dancer with many partners. J. Cell Sci..

[60] Maul R.W., Scouten Ponticelli S.K., Duzen J.M., Sutton M.D. (2007). Differential binding of *Escherichia coli* DNA polymerases to the β-sliding clamp. Mol. Microbial..

[61] Johnson D.E., Takahashi M., Hamdan S.M., Lee S.-J., Richardson C.C. (2007). Exchange of DNA polymerases at the replication fork of bacteriophage T7. Proc. Natl. Acad. Sci. USA.

[62] Hacker K.J., Alberts B.M. (1994). The rapid dissociation of the T4 DNA polymerase holoenzyme when stopped by a DNA hairpin helix. A model for polymerase release following the termination of each okazaki fragment. J. Biol. Chem..

[63] Cha T.-A., Alberts B.M. (1989). The bacteriophage t4 DNA replication fork. Only DNA helicase is required for leading strand DNA synthesis by the DNA polymerase holoenzyme. J. Biol. Chem..

[64] Dong F., Weitzel S.E., Von Hippel P.H. (1996). A coupled complex of T4 DNA replication helicase (gp41) and polymerase (gp43) can perform rapid and processive DNA strand-displacement synthesis. Proc. Natl. Acad. Sci. USA.

[65] Delagoutte E., von Hippel P.H. (2001). Molecular mechanisms of the functional coupling of the helicase (gp41) and polymerase (gp43) of bacteriophage T4 within the DNA replication fork. Biochemistry.

[66] Ishmael F.T., Trakselis M.A., Benkovic S.J. (2003). Protein-protein interactions in the bacteriophage T4 replisome the leading strand holoenzyme is physically linked to the lagging strand holoenzyme and the primosome. J. Biol. Chem..

[67] Stano N.M., Jeong Y.-J., Donmez I., Tummalapalli P., Levin M.K., Patel S.S. (2005). DNA synthesis provides the driving force to accelerate DNA unwinding by a helicase. Nature.

[68] Manosas M., Spiering M.M., Ding F., Croquette V., Benkovic S.J. (2012). Collaborative coupling between polymerase and helicase for leading-strand synthesis. Nucl. Acids Res..

[69] Lionnet T., Spiering M.M., Benkovic S.J., Bensimon D., Croquette V. (2007). Real-time observation of bacteriophage T4 gp41 helicase reveals an unwinding mechanism. Proc. Natl. Acad. Sci. USA.

[70] Salinas F., Benkovic S.J. (2000). Characterization of bacteriophage T4-coordinated leading-and lagging-strand synthesis on a minicircle substrate. Proc. Natl. Acad. Sci. USA.

[71] Yang J., Trakselis M.A., Roccasecca R.M., Benkovic S.J. (2003). The application of a minicircle substrate in the study of the coordinated T4 DNA replication. J. Biol. Chem..

[72] Jing D., Beechem J.M., Patton W.F. (2004). The utility of a two-color fluorescence electrophoretic mobility shift assay procedure for the analysis of DNA replication complexes. Electrophoresis.

[73] Jing D.H., Dong F., Latham G.J., von Hippel P.H. (1999). Interactions of bacteriophage t4-coded primase (gp61) with the t4 replication helicase (gp41) and DNA in primosome formation. J. Biol. Chem..

[74] Manosas M., Spiering M.M., Zhuang Z., Benkovic S.J., Croquette V. (2009). Coupling DNA unwinding activity with primer synthesis in the bacteriophage T4 primosome. Nat. Chem. Biol..

[75] Lee J.-B., Hite R.K., Hamdan S.M., Xie X.S., Richardson C.C., Van Oijen A.M. (2006). DNA T4 primase acts as a molecular brake in DNA replication. Nature.

[76] Pandey M., Syed S., Donmez I., Patel G., Ha T., Patel S.S. (2009). Coordinating DNA replication by means of priming loop and differential synthesis rate. Nature.

[77] Kadyrov F.A., Drake J.W. (2001). Conditional coupling of leading-strand and lagging-strand DNA synthesis at bacteriophage T4 replication forks. J. Biol. Chem..

[78] Trakselis M.A., Roccasecca R.M., Yang J., Valentine A.M., Benkovic S.J. (2003). Dissociative properties of the proteins within the bacteriophage T4 replisome. J. Biol. Chem..

[79] Trakselis M.A., Alley S.C., Abel-Santos E., Benkovic S.J. (2001). Creating a dynamic picture of the sliding clamp during T4 DNA polymerase holoenzyme assembly by using fluorescence resonance energy transfer. Proc. Natl. Acad. Sci. USA.

[80] Nelson S.W., Kumar R., Benkovic S.J. (2008). Rna primer handoff in bacteriophage T4 DNA replication the role of single-stranded DNA-binding protein and polymerase accessory proteins. J. Biol. Chem..

[81] Yang J., Nelson S.W., Benkovic S.J. (2006). The control mechanism for lagging strand polymerase recycling during bacteriophage T4 DNA replication. Mol. Cell.

[82] Carver T.E., Sexton D.J., Benkovic S.J. (1997). Dissociation of bacteriophage t4 DNA polymerase and its processivity clamp after completion of okazaki fragment synthesis. Biochemistry.

[83] Tougu K., Marians K.J. (1996). The interaction between helicase and primase sets the replication fork clock. J. Biol. Chem..

[84] Kurth I., Georgescu R.E., O’Donnell M.E. (2013). A solution to release twisted DNA during chromosome replication by coupled DNA polymerases. Nature.

[85] Yuan Q., McHenry C.S. (2014). Cycling of the *E. coli* lagging strand polymerase is triggered exclusively by the availability of a new primer at the replication fork. Nucl. Acids Res..

[86] Geertsema H.J., van Oijen A.M. (2013). A single-molecule view of DNA replication: The dynamic nature of multi-protein complexes revealed. Curr. Opin. Struct. Biol..

[87] Chen D., Yue H., Spiering M.M., Benkovic S.J. (2013). Insights into okazaki fragment synthesis by the T4 replisome the fate of lagging-strand holoenzyme components and their influence on Okazaki fragment size. J. Biol. Chem..

